# Accidentally ingested wooden toothpick, perforation of a sigmoid diverticulum and mimicking acute colonic diverticulitis: A case report and literature review

**DOI:** 10.1016/j.ijscr.2023.107945

**Published:** 2023-02-21

**Authors:** Giuseppe Evola, Giulia Impellizzeri, Elia Pulvirenti, Maria D'Angelo, Martina Reina, Giuseppe Angelo Reina

**Affiliations:** a General and Emergency Surgery Department, Garibaldi Hospital, Piazza Santa Maria di Gesù 5, 95124 Catania, Italy; b General Surgery Department, Santissimo Salvatore Hospital (ASP Catania), Paternò, Catania, Italy

**Keywords:** Wooden toothpick, Ingested foreign bodies, Gastrointestinal perforation, Acute abdomen, Emergency surgery, Case report

## Abstract

**Introduction and importance:**

Ingested wooden toothpick (WT) represents a rare cause of acute abdomen. Preoperative diagnosis of ingested WT is a challenge because of its unspecific clinical presentation, the low sensitivity rate of radiological investigations and the patient's inability to often recall the event of swallowing a WT. Surgery represents the main treatment in case of ingested WT-induced complications.

**Case presentation:**

A 72-year-old Caucasian male presented to the Emergency Department with a two-day history of left lower quadrant (LLQ) abdominal pain, nausea, vomiting and fever. Physical examination revealed LLQ abdominal pain and rebound tenderness with muscle guarding. Laboratory tests reported high levels of C-reactive protein and neutrophilic leukocytosis. Abdominal contrast-enhanced computed tomography (CECT) showed colonic diverticulosis, wall thickening of the sigmoid colon, pericolic abscess, regional fatty infiltration, a suspicion of sigmoid perforation secondary to a foreign body. The patient underwent diagnostic laparoscopy: a sigmoid diverticular perforation caused by an ingested WT was noticed and a laparoscopic sigmoidectomy with end-to-end Knight-Griffen colorectal anastomosis, partial omentectomy and protective *loop ileostomy* were performed. The postoperative course was uneventful.

**Clinical discussion:**

The ingestion of a WT represents a rare but potentially fatal condition which may cause GI perforation with peritonitis, abscesses and other rare complications if it migrates out of the GI tract.

**Conclusion:**

Ingested WT may cause serious GI injuries with peritonitis, sepsis or death. Early diagnosis and treatment are crucial for reducing morbidity and mortality. Surgery is mandatory in case of ingested WT-induced GI perforation and peritonitis.

## Highlights

• A wooden toothpick (WT)-induced perforation of a sigmoid diverticulum is a rare cause of acute abdomen.
• Diagnosis of WT-induced gastrointestinal (GI) perforation is a challenge because of the absence of specific clinical presentation.
• WT impaction can be difficult to diagnose through radiology.
• Radiology plays an important role in the diagnosis of WT-induced GI perforation
• Surgery represents the main treatment in case of WT-induced open GI perforation and peritonitis.

## 1 Introduction

Ingestion of a wooden toothpick (WT) represents a rare event mainly favored by psychiatric conditions, dementia, young or old age, consuming foods containing WT, rapid eating, alcoholic beverage, and carriage of denture [1]. Although the majority of the ingested foreign bodies pass the gastrointestinal (GI) tract spontaneously without complications, 10 % to 20 % of them require endoscopic removal and 1 % or less requires surgical intervention [2]. Reported complications after foreign body (FB) ingestion are obstruction, perforation, hemorrhage, fistula formation and sepsis [3]. WT ingestion carries a greater risk of perforation due to its sharp points and it is difficult to diagnose because most patients do not recall swallowing one, symptoms and signs are nonspecific and a WT has a radiolucent character [4]. A rare case of accidentally ingested WT causing perforation of a sigmoid diverticulum and mimicking acute colonic diverticulitis is presented with review of the literature in accordance with SCARE 2020 criteria [5]. The purpose of this case report is to remember that WT ingestion is a rare cause of acute abdomen that may require emergency surgery.

## 2 Presentation of case

**Fig. 1:**
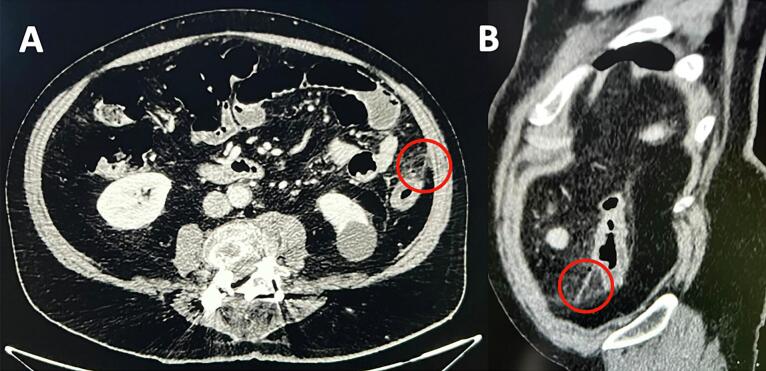
**A**, **B** Abdominal CECT howing a suspicion of sigmoid perforation secondary to a foreign body (red circle). A transverse, B sagittal view. (For interpretation of the references to colour in this figure legend, the reader is referred to the web version of this article.)

**Fig. 2:**
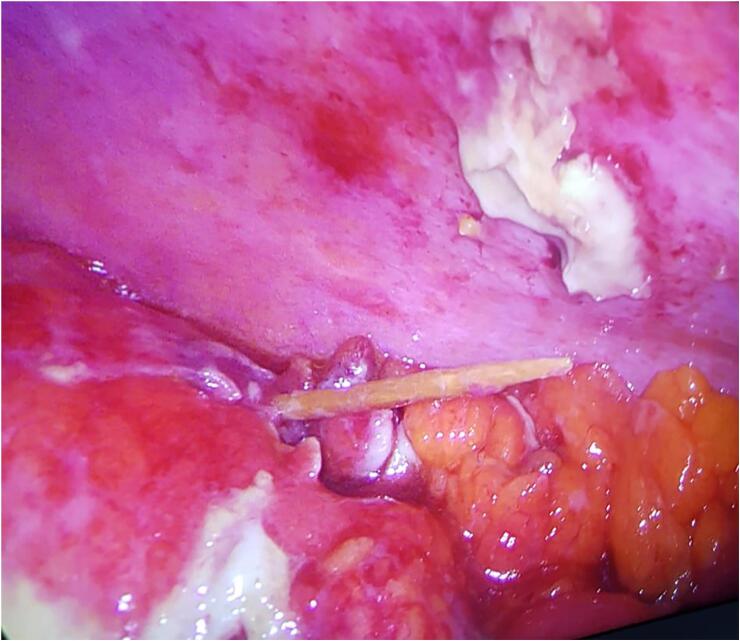
Sigmoid diverticular perforation caused by a WT: operative findings.

**Fig. 3:**
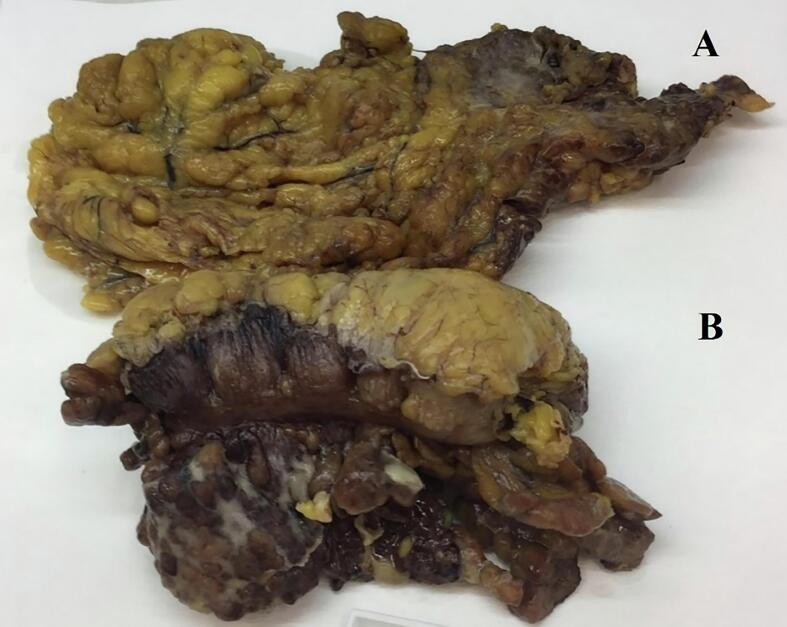
The surgical specimen consisted of 15 × 9 cm of omentum fringe (A) and of 10 cm of sigmoid colon bearing the perforated diverticulum (B).

**Fig. 4:**
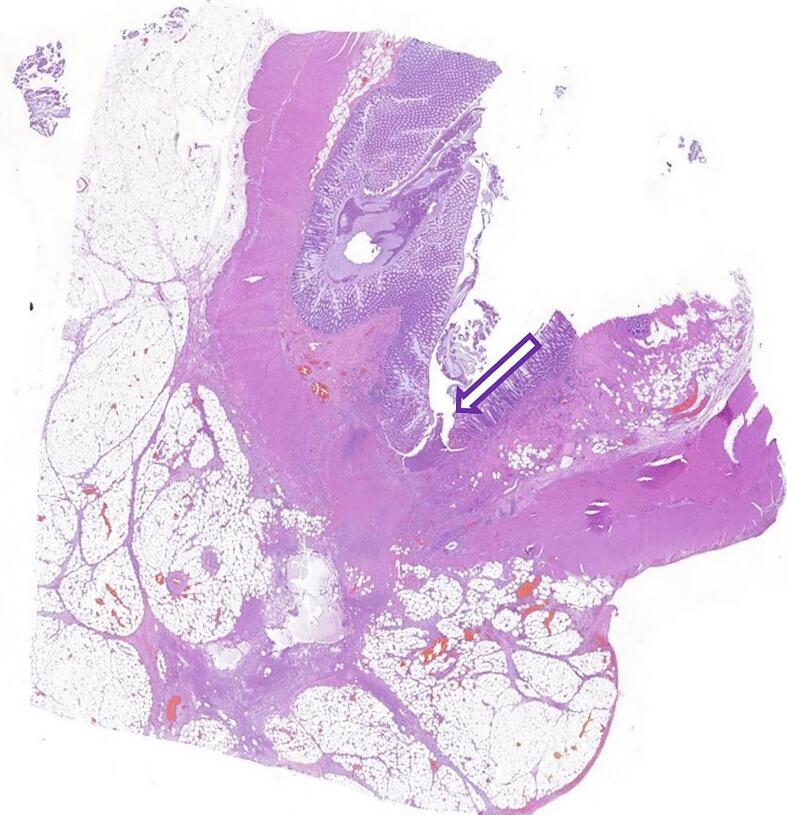
Photomicrograph section of discontinuity of colonic mucosa (purple arrow), haematoxylin and eosin (original magnification × 20). (For interpretation of the references to colour in this figure legend, the reader is referred to the web version of this article.)

A 72-year-old Caucasian male presented to the Emergency Department with a two-day history of left lower quadrant (LLQ) abdominal pain, nausea, vomiting and fever (38 °C); others vital signs were normal. His past medical history included arterial hypertension, colonic diverticulosis, and appendectomy. The patient, wearing dentures, was on hypertensive medications for ten years and referred habit on smoking but denied alcohol consumption; his familial medical history was normal. He was retired from the work, married and of medium socio-economic status. Physical examination revealed mild abdominal distention, LLQ abdominal pain on superficial and deep palpation with obvious muscle guarding and rebound tenderness, hypoactive bowel sound. Laboratory tests reported high levels of C-reactive protein (90.5 mg/L) and neutrophilic leukocytosis (WBC 16.700 10^3^/μL). The patient was initially managed with fluids, intravenous broad-spectrum antibiotics, antipyretic drugs and bowel rest. After an abdominal ultrasound exam, the patient was evaluated by abdominal contrast-enhanced computed tomography (CECT) which confirmed colonic diverticulosis and revealed wall thickening of the sigmoid colon, pericolic abscess, regional fatty infiltration, a suspicion of sigmoid perforation secondary to a FB (Fig. 1A,B). The patient, after understanding the severity of his medical condition and accepting surgery, was taken emergently to the operating room by experienced general surgeons for diagnostic laparoscopy under general anesthesia. After induction of pneumoperitoneum with the Veress needle and placement of four trocars (two 12-mm trocars in the umbilical region and right iliac fossa and two 5-mm trocars in the right hypochondrium and right flank) we explored the peritoneal cavity with evidence of a thickened and fibrin-covered omentum tenaciously adhering to the left parieto-colic douche. We performed lysis of omental adhesions with evidence of a sigmoid diverticular perforation caused by a WT (Fig. 2) and covered by the same omentum, wall thickening of the sigmoid colon and pericolic inflammation. A laparoscopic sigmoidectomy with end-to-end Knight-Griffen colorectal anastomosis, a partial omentectomy and a protective *loop ileostomy* were performed. After peritoneal washing and aspiration, a laminar perianastomotic drain was placed. Patient was given an IV injection of Amoxicillin/Clavulanate 2 g twice daily and Metronidazole 500 mg thrice daily for five days and a SC injection of enoxaparin sodium 4.000 IU once daily for 21 days. The postoperative course was uneventful, laboratory tests were unremarkable and abdominal drain was removed on the 5th postoperative day. The patient, who reported consuming food containing WT two weeks before emergency surgery, was discharged on the 6th postoperative day in a stable condition. The surgical specimen, fixed in formalin (Fig. 3), consisted of 10 cm of sigmoid colon bearing the perforated diverticulum and of 15 × 9 cm of omentum fringe covered with fibrin. Histopathological examination confirmed the mucosal discontinuity and revealed the presence of an abscessed inflammatory infiltrate in the thickness of the colonic wall and in the perivisceral adipose tissue (Fig. 4). Stapled closure of loop ileostomy was performed three months after laparoscopic sigmoidectomy. The patient tolerated the advice provided and after a follow-up of six months is asymptomatic.

## 3 Discussion

This rare clinical case describes a delayed presentation of WT-induced perforation of a sigmoid diverticulum leading to acute abdomen. FB ingestion represents a common problem in everyday emergency clinical practice. Foreign bodies include a wide range of materials, from biological ones such as bones [6] and trichobezoar [7] to artificial materials such as shots and batteries. Although most swallowed foreign bodies generally travel through the GI tract without any problem, some of them can cause complications like as impaction, perforation (<1 % of patients), bleeding, obstruction, fistula formation and sepsis [4]. Ingested WT represents a rare, potentially fatal condition which may cause GI perforation with peritonitis, abscesses and other rare complications if it migrates out of the GI tract [8]. Ingested WT carries a greater risk of GI perforation and internal organ injury than other foreign bodies due to its pointed shaped: according to a recent review, the incidence of WT-related gut perforation is as high as 80 % [9]. The main risk factors associated with WT ingestion include male gender, being a child or elderly person, dull palatal sensation due to dentures, alcohol or drug use, habitual chewing of WT, consuming foods containing WT, rapid eating, psychiatric disorders, dementia [4], [9]. Although all the portions of GI tract can be sites of WT lodgment or perforation, the most common areas are those controlled by sphincters, physiological narrowing and acute flexures like as duodenum (23 %) stomach (20 %), small intestine (18 %) and sigmoid colon (16 %) [9], [10]. Other GI sites where WT impaction is most likely include zones with adhesions, areas containing a diverticular process (as in our case) or surgical anastomoses [10], [11]. After GI perforation the WT can migrate into adjacent or distant organs including pleura, liver, peritoneal or retroperitoneal space, pericardium, urinary bladder, great vessels, pancreas, ureter, hepatoduodenal ligament, lungs, and kidneys [4], [8]. Although an early diagnosis and treatment are crucial for reducing morbidity and mortality, a correct diagnosis may be difficult partly due to the absence of specific clinical presentation and laboratory tests, the low sensitivity rate of radiological investigations but mostly due to patient's inability to recall the event of swallowing a WT. The suspected diagnosis is usually acute appendicitis, acute diverticulitis (as in our case) or perforation of a hollow viscus. In a systemic review 54 % of patients were unaware of the event and majority (67 %) of them ingested the WT during food intake, 85 % of patients reported habit of chewing toothpicks [9]. The time from WT ingestion to clinical presentation is long (more than two weeks in 50 % of cases, months or even years in some cases) so patients do not usually associate their symptoms to WT ingestion [12]. Clinical symptoms and signs vary from abdominal pain with or without fever, nausea or diarrhea to focal or diffuse peritonitis or intra-abdominal abscess. Among diagnostic tests, endoscopy appears to have the highest sensitivity (72.1 %) in WT identification; standard X-ray studies (sensitivity of 5–15 %) or ultrasound imaging (sensitivity of 32.6 %) usually fail in identifying the ingested WT because of its radiolucent nature and small diameters [9], [13]. CECT is able to localize the WT with a sensitivity of 42.6 % [9] but can also identify the site of GI perforation, localized pneumoperitoneum, the extent of intra-abdominal inflammation with or without abscess, the migration of WT into adjacent organs. A careful interpretation of CECT, using reformatted coronal and sagittal images, may be helpful in WT identification [14]. Magnetic resonance imaging may be a better diagnostic tool for detecting WT [15]. Because of the low sensitivity of radiological imaging, definitive diagnosis can be mostly obtained with surgery. In our case report the ingested WT was detected on abdominal CECT. Upper gastrointestinal endoscopy and ultrasound examination are recommended in asymptomatic patients who are aware of the WT ingestion and seek medical advice within 24–48 h [9]: endoscopy is the most effective technique for identifying and removing the WT embedded in the proximal or in the distal GI tract. If the WT is located in the small intestine a diagnostic laparoscopy should be performed. Surgical treatment is mandatory in the presence of ingested WT-induced complications like as GI perforation, peritonitis, abscesses, fistulas, intractable bleeding or WT migration to adjacent extra-GI structures [16]. If there is evidence of WT-induced GI perforation or peritonitis the laparotomic or laparoscopic treatment can vary from suture to intestinal resection based on the contamination of the peritoneal cavity and the severity of organ injury. Impaction and perforation of GI tract from ingested WT should be considered in the differential diagnosis of acute abdominal pain because of WT ingestion is a life-threatening event with a mortality rate of 9.6 %–18 % [9], [17].

## 4 Conclusion

Ingested WT is a rare event which may cause serious GI injuries with peritonitis, sepsis or death. When risk factors for FB ingestion are present, physicians should be aware of the possibility of FB ingestion like as rare cause of acute abdomen Imaging, endoscopy and/or laparoscopy are helpful in detecting WT ingestion and its complications. Surgery is mandatory in case of WT-induced GI perforation and peritonitis.

## Ethical approval

Ethical approval has been exempted by our institution because this is a case report and no new studies or new techniques were carried out.

## Sources of funding

Not applicable.

## Consent

Written informed consent was obtained from the patient, for publication of this case report and accompanying images. A copy of the written consent is available for review by the Editor-in-Chief of this journal on request.

## Provenance and peer review

Not commissioned, externally peer-reviewed.

## Guarantor

The guarantor for this case report is Giuseppe Evola.

## Registration of research studies

Not applicable.

## CRediT authorship contribution statement

Giuseppe Evola: Drafting the manuscript, literature research.

Giulia Impellizzeri: Operated on the patient, drafting the manuscript.

Elia Pulvirenti: Operated on the patient, drafting the manuscript.

Maria D’Angelo: Drafting the manuscript, literature research.

Martina Reina: Drafting the manuscript, literature research.

Giuseppe Angelo Reina: Operated on the patient, revising the manuscript.

## Declaration of competing interest

Not applicable.
